# A New Strategy on Designing Fluxes for Aluminum Alloy Melt Refinement

**DOI:** 10.3390/ma16062322

**Published:** 2023-03-14

**Authors:** Guoqing Zhang, Weihong Lu, Xiaocong Wu, Bo Yang, Yapeng Tan, Zhengbing Xu, Hongqun Tang, Jianmin Zeng, Junsheng Wang

**Affiliations:** 1 State Key Laboratory of Featured Metal Materials and Life-Cycle Safety for Composite Structures, Guangxi University, Nanning 530004, China; 2 Centre of Ecological Collaborative Innovation for Aluminum Industry in Guangxi, Guangxi University, Nanning 530004, China; 3 Key Laboratory of High-Performance Structural Materials and Thermo-Surface Processing (Guangxi University), Education Department of Guangxi Zhuang Autonomous Region, Nanning 530004, China; 4 Advanced Research Institute of Multidisciplinary Science, Beijing Institute of Technology, Beijing 100081, China

**Keywords:** AA6111 alloys, melt treatment, flux, high-temperature contact angle

## Abstract

With the aim of obtaining a refining flux that is stable and provides effective refining of aluminum melt, a new strategy of designing the flux composition has been proposed. Ten fluxes were designed, by selecting ten molten salt compounds according to their thermophysical parameters, physical properties, and thermodynamic analysis. The melting points of the ten fluxes, and the phases transformation of the fluxes after melting, were studied by DSC and XRD, respectively. The contact angles between four groups of fluxes and alumina at refinement temperatures were studied, and the effect of refinement was characterized by a metallographic microscope. The process of the fluxes removing inclusions and degassing was analyzed thermodynamically. The research findings indicate that flux #10 (11.0 wt.%NaF, 29.5 wt.%NaCl, 46.5 wt.%Na_2_CO_3_, 3.0 wt.%CaF_2_, 10.0 wt.%Na_3_AlF_6_) has a melting point (562.2 °C) below the refining temperature. At the refining temperature (760 °C), flux #10 has the lowest contact angle, of 12.78°. In addition, compared to that of flux STJ–A3, currently used in practice, flux #10 has a better refining effectiveness, with the pores and inclusions content of the sample being reduced to 1.11% from 2.96%.

## 1. Introduction

Aluminum alloys are widely used because of their light weight [1], high specific strength [2], high ductility [3], corrosion resistance, low price, and excellent manufacturability [4]. However, nonmetallic inclusions may be introduced from the raw materials used in the extractive metallurgical processes, from the refractory materials and the atmosphere during aluminum production, and even from the refining processes [5]. Nonmetallic inclusions (mainly aluminum oxide), including undesired gases (hydrogen), and oxides, borides, nitrides, carbides, and chlorides in aluminum may cause serious defects [6,7,8]. These inclusions cluster, causing the formation of pores in aluminum [9]. To remove these nonmetallic inclusions, scholars have developed a variety of methods, such as sedimentation, ultrasonication, bubble floatation, filtration [10,11], electromagnetic stir technology [12,13,14], flux refining [15], and so on. Among them, flux refinement is widely used in industrial production, because of its low cost and operational simplicity.

In the past decades, the development of solid fluxes for aluminum alloy melt refinement has mainly focused on the NaCl–KCl system, as it was found that equimolar NaCl and KCl form a low-temperature stable eutectic at 665 °C [16,17,18,19,20,21,22,23]. This led to a boom in the development and basic research of NaCl–KCl based fluxes. As Utigard and Besson et al. [19,24,25] proposed that the addition of NaF, Na_3_AlF_6_, CaF_2_, KF, or Na_2_SiF_6_ to NaCl–KCl would reduce the wetting angle between the flux and solid inclusions, and improve the inclusion-removal property of the flux. Li et al. [18] studied the wetting behavior of the flux and oxide film using a mixture of NaCl and KCl. Majidi et al. [26] proposed a flux by adding Na_2_SiF_6_ and CaF_2_ to the NaCl–KCl mixture, and studied the effect of the flux melting temperature on the refining process of molten aluminum alloy. Tenorio et al. [16] designed a flux with 5% NaF, KF, and CaF_2_ in mixtures of NaCl and KCl, and studied the effect on flux viscosity of the addition of different fluorides. Chen et al. [27] studied the effect of NaCl–KCl flux and NaCl–KCl flux with added KF, in fluxing treatment of A356.2 alloy, and found that only the NaCl–KCl flux reduced the relative porosity of the A356.2 alloy. Wan et al. [28] designed a flux by adding KF and K_3_AlF_6_ or KAlF_4_ to equimolar NaCl–KCl, which improved the separating effect of the flux from the melt surface, and the stripping ability of the oxide layer for the flux. Hiraki et al. [29] pointed out that the addition of AlCl_3_ to equimolar NaCl–KCl could potentially remove Ca, Ce, Dy, Dg, Ho, La, Li, Mg, and Sr. The design of the fluxes in these studies was limited to the choice of chlorine and fluorine salts, and the design process was mostly orthogonal and the choice of composition was arbitrary.

In recent years, the development of fluxes has not stopped. Achebo et al. [30] developed a new flux based on NaCl–CaCl_2_–CaF_2_–Na_3_AlF_6_, by applying the Hadamard multivariate chemical composition model. Tkacheva et al. [31] found that a potassium cryolite-based flux (the KF–AlF_3_ and KF–NaF (10 wt.% AlF_3_) molten mixtures with CR = 1.3–1.5) could play a better refining role in aluminum alloy production. Ni et al. [32] designed a new JDN–Ι aluminum alloy flux, containing rare earth compounds, their experimental results indicated that it had a great effect on the secondary dendrite arm spacing (DAS) of A356 alloy. Tsunekawa et al. [33] developed a flux by adding AlF_3_, K_3_AlF_6_, etc., to KCl, but only a range of components for each molten salt was given. Widyantoro et al. [34] developed a Na_2_SO_4_ and NaCl based flux, and found that it could remove oxide inclusions efficiently. However, the limitations of molten salt selection and the arbitrary choice of the salt composition, resulted in a single functional flux and a high cost in terms of economy and time, these problems have not been addressed in the flux design progress of these scholars. Thus, it is essential to find a method for designing flux compositions that is efficient and takes all the most molten salts into account.

In this study, a new comprehensive strategy on designing flux composition has been proposed. Firstly, thermophysical parameters, such as the melting points and phase composition of fluxes, were characterized through DSC and XRD, respectively. Secondly, the high-temperature contact angle of the flux to the alumina was tested. Finally, the spontaneity of the flux to adsorb inclusions in the melt was analyzed thermodynamically. Ten fluxes were designed, by selecting ten molten salt compounds according to their thermophysical parameters (melting point), and the eutectic points of the molten salt systems. These ten fluxes were used to refine the aluminum melt, and the refining efficiency was characterized by metallography, compared with that of a selected flux currently used in practice.

## 2. Materials and Methods

The most commonly used main components of fluxes in aluminum melt refining are chlorides and fluorides, such as NaCl, KCl, NaF, AlF_3_, etc. Others, such as Na_3_AlF_6_, Na_2_CO_3_, K_2_CO_3_, etc., are often added to fluxes. The chlorides and fluorides are selected as the base of the flux due to their low surface tension and good coverage performance [19]. Na_2_CO_3_, Na_2_SO_4_, and NaNO_3_ are used as heating agents in the flux. CaF_2_ and Na_3_AlF_6_ can improve the wettability between the flux and inclusions, and generate molten colloid to adsorb inclusions in the aluminum melt.

Usually, it is of significance that the melting point and density of flux are lower than those of the aluminum melt, so that the flux can melt below the melting point of the aluminum alloy and can float up to the aluminum melt surface. Table 1 shows the most commonly used salt compounds and their physical properties [19]. Some of the salt compounds show higher melting points than those of aluminum alloys. But, in general, the fluxes used in practice are the mixtures of several salt compounds, through which the melting point of fluxes will be lowered below those of aluminum alloys. As known from phase diagram theory, binary or multicomponent eutectic, while not normally interacting to create a new chemical substance, inhibit the crystallization phase of one another at certain ratios, resulting in a system with a lower melting point than any of the single components, from which the strategy of this work stemmed.

Table 2 lists the eutectic point compositions and eutectic temperatures of selected and commonly used fundamental flux systems. Most of the salt systems have lower eutectic temperatures than the melting point of aluminum alloys. This work selected some of the salt systems with suitable eutectic temperatures as the basic compositions of an alternative flux.

### 2.1. The Sample Preparation

The salt compositions used in this work are shown in Table 3, with purities ≥ 99.0%. All the salts were put into a blast drying oven and dried for 24 h at 120 °C. Salt samples were weighed according to the sample compositions listed in Table 3, and then were put into an agate grinding bowl for full grinding and mixing. The salt mixtures, i.e., fluxes, were put into an alumina crucible with a lid and kept in a muffle furnace at 760 °C for 6 h, for melting and further mixing. After melting, these mixed salt fluxes were dried in a blast drying oven at 120 °C for 24 h. Finally, the salt fluxes were ground into powder and wrapped in aluminum foil for subsequent experiments.

AA6111 aluminum alloy has good corrosion resistance and plasticity, moderate strength, and excellent cold working performance, and is widely used in building decoration materials, manufacturing vehicle parts, ships, etc. AA6111 alloy (95 wt.%) and aluminum scrap (5 wt.%) are used as the experimental raw materials for refinement in this work, and the specific composition of AA6111 alloy is given in Table 4 (GB/T 3190–2008). The refining efficiency of all the fluxes, as shown in Table 3, on AA6111 aluminum melts are investigated, compared with that of the STJ–A3 flux, currently used in practice.

### 2.2. Methods

The melting points of samples #1 to 10 were determined using a differential scanning calorimeter (NETZSCH 404 C). The sample powder of 10–15 mg was weighed using a balance with a readability of ±0.01 mg, and the samples were tested in an argon environment. The measurement crucible and reference crucible were platinum crucibles with lids.

X-ray diffraction (XRD) analysis of the mixed flux powder was carried out using a Rigaku D/MAX 2500 V X-ray diffractometer, at a voltage of 40 kV and a current of 150 mA. The reaction equilibrium constant calculations in this study were all performed by the HSC Chemistry 6.0 software.

An OCA25–HTV 1800 was used for the high-temperature contact angle test. According to Gyarmati [15], Li [18], Chen [27], Wan [28], and Fu et al. [9], the most common oxide inclusions within the aluminum melt are aluminum oxide. Because the aluminum oxide inclusions within the aluminum melt show an amorphous state, taking the form of strings and films, it is hard to study the contact angle between the fluxes and the actual aluminum oxide. Therefore, the alumina plate was chosen as the alternative material of aluminum oxide inclusions, to carry out the contact angle test, because they have similar surface properties. The solid sample was processed into a cylindrical shape and placed on an alumina ceramic plate, and the whole sample was fed into the furnace tube, and heated through the high-temperature furnace until the sample melted. Photos of the melted state were taken by a video system and then analyzed. The sample was heated from room temperature to 760 °C with a heating rate of 5 K/min. The contact angle was calculated according to the sessile drop method, which measures the static contact angle. Based on the recognized images, the software automatically finds the baseline (solid–liquid contact surface), fits the captured droplet shape according to the ellipse method, and then calculates the contact angle between the solid–liquid and gas–liquid phases, if available.

The standard burden, of 1.5 kg AA6111 alloy, was melted in a graphite crucible resistance furnace. After removing the surface oxide film, the prepared flux (1.0% the weight of alloy) was pressed into the bottom of the aluminum melt using a bell jar, and the melt was stirred lightly for 5 min. After the flux melting, the bell jar was removed and the AA6111 melt was sedimented for 20 min. Finally, the surface slag was removed and poured into the mold. The tools and raw materials used in the above experiments were dried at 200 °C (24 h) in a blast drying oven before use.

Usually, the refining effectiveness of flux is available from the pores and oxide inclusions areas in aluminum alloy through metallography. The AA6111 castings were cut into small cylinders by wire cutting and ground with 400, 600, 800, 1000, 1500, and 2000 grit paper, and then polished with 5000 grit diamond polishing paste. The polished samples were observed using a metallographic microscope under a 10 X objective lens, and photographed at randomly selected locations, in principle, the total area of the images should cover more than 70% of the total area of the polished surface, and the area of the pores and inclusions was calculated using the “segmentation” function in the software, after the filming was completed. The pores and oxide inclusions ratio was obtained from Equation (1):

(1) $$n=\frac{S}{S_{n}}$$

where *n* is the percentage of the pores and inclusions, *S* is the area of the pores and inclusions, and *S_n_* is the total area of the images.

## 3. Results and Discussion

### 3.1. Thermophysical Parameters of the Fluxes

#### 3.1.1. The Melting Points of Fluxes

**Figure 1:**
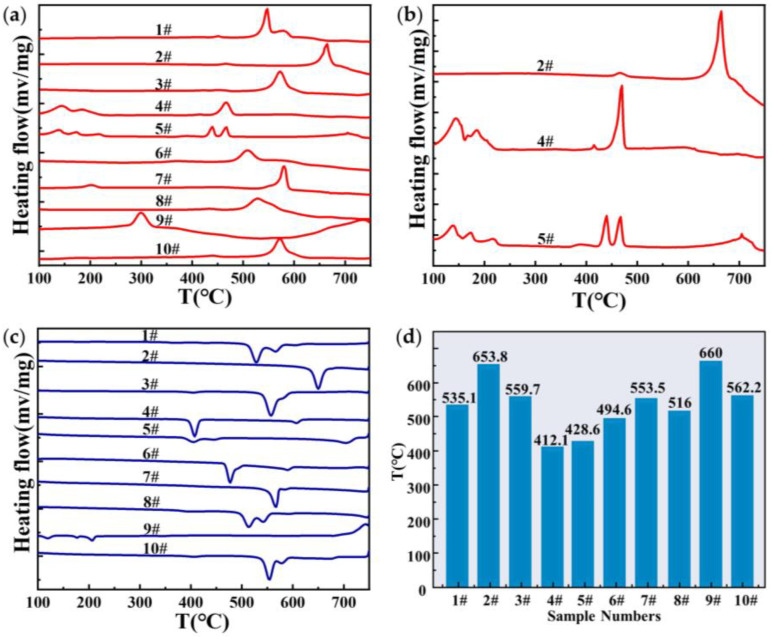
The results of DSC: (**a**) DSC heating curves of fluxes #1–#10, with the rate of 5 K/min, in an atmosphere of nitrogen; (**b**) enlarged view of the heating curves of fluxes #2, #4, and #5; (**c**) DSC cooling curves of fluxes #1–#10, with the rate of 5 K/min, in an atmosphere of nitrogen; (**d**) the melting points of the fluxes.

Figure 1 shows the DSC heating and cooling curves for samples #1 to 10, and the melting points of the fluxes.

As shown in Figure 1a, the heating curves of fluxes #3, #6, #7, #8, and #10 showed only a single endothermic peak. Fluxes #1, #2, #4, #5, and #9 showed multiple endothermic peaks in the heating curves. The presence of multiple endothermic peaks in the heating curve indicates that there are multiple thermal effects during sample heating, while the presence of a single endothermic peak indicates that there is only one thermal effect during sample heating [43,44].

In this study, the melting point of the flux was defined as the onset point [45] of the first stable endothermic peak in the heating curve [46] (i.e., there is a corresponding thermal effect peak in the cooling curve). Thus, the melting points of fluxes #1, #3, #6, #8, and #10 are the onset points of the endothermic peaks. Several endothermic peaks were present in the heating curves of fluxes #4, #5, and #7 at 100–250 °C. According to Table 3, fluxes #4 and #5 contain MgCl_2_, and flux #7 contains K_2_CO_3_, the three compounds have extremely high water absorption. So, it is inferred that the endothermic peak is caused by sample dehydration. As a consequence, the melting points of fluxes #4 and #5 are the onset point of the endothermic peak that appears at 400–500 °C, as shown in Figure 1b. For flux #2, a small endothermic peak appears at 400–500 °C. Due to the high temperature, and the fact that the corresponding thermal effect peak does not appear in the cooling curve, it is inferred that it is caused by an irreversible reaction. As a result, the melting point of flux #2 should be the onset point of the endothermic peak appearing at 600–700 °C, as shown in Figure 1b. In the heating curve of flux #9, there is an endothermic peak at 300 °C, but it does not correspond to the cooling curve (shown in Figure 1b). It is conjectured that sodium nitrate undergoes a second-order (order–disorder) phase transition at this temperature [47]. Therefore, the melting point of flux #9 should be the onset point of the endothermic peak appearing at 600–700 °C. All the melting points of the fluxes, as shown in Figure 1d, will be further confirmed in the high-temperature contact angle experiments.

#### 3.1.2. The Phase Compositions of the Fluxes

**Figure 2:**
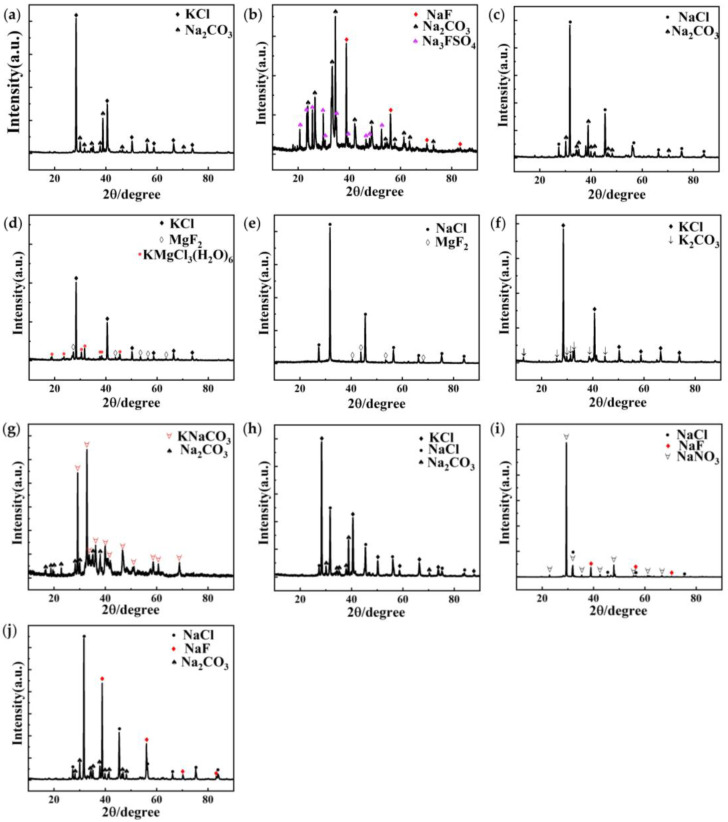
XRD spectra of the fluxes: (**a**) #1; (**b**) #2; (**c**) #3; (**d**) #4; (**e**) #5; (**f**) #6; (**g**) #7; (**h**) #8; (**i**) #9; (**j**) #10.

The XRD spectra of the fluxes is shown in Figure 2. Figure 2a shows the XRD spectrum of flux #1. The main components of flux #1 are KCl and Na_2_CO_3_. After melting, no other phases were found in the XRD diffraction spectrum, indicating that the composition of the flux after melting is stable, and there is no reaction between the main components.

Figure 2b shows the XRD spectrum of flux #2. The main components of flux #2 are Na_2_SO_4_ and Na_2_CO_3_, the phases shown in the figure are NaF, Na_2_CO_3_, and Na_3_FSO_4_, NaF, and Na_3_FSO_4_ are produced in flux #2 after the reaction. Their reactions are shown in the following response equations [48]:

(2) $${3\mathrm{Na}}_{2}{\mathrm{SO}}_{4}+2{\mathrm{Na}}_{3}{\mathrm{AlF}}_{6}=12\mathrm{NaF}+{\mathrm{Al}}_{2}{\left({\mathrm{SO}}_{4}\right)}_{3}$$

(3) $$\mathrm{NaF}+{\mathrm{Na}}_{2}{\mathrm{SO}}_{4}={\mathrm{Na}}_{3}{\mathrm{FSO}}_{4}$$

The calculated reaction equilibrium constant of Equation (2) is *K*_eq_ = 9.132·10^−30^, and Equation (3) is confirmed in the NaF–Na_2_SO_4_ system, and Na_3_FSO_4_ exists in the NaF–Na_2_SO_4_ system. The reaction corresponds to the small endothermic peak of flux #2 at 700 °C (as shown in Figure 1b). The heating curve of flux #2 moves in the negative direction of the y-axis after the reaction occurs, indicating that the reaction releases a quantity of heat.

Figure 2c,f,h–j show the XRD spectra of fluxes #3, #6, #8, #9, and #10, respectively. All these fluxes have no change in the physical phase before and after melting. The main components of flux #4 in Figure 2d are KCl and MgCl_2_, and then its composition changed to KCl, MgF_2_, and KMgCl_3_(H_2_O)_6_ after melting. The reaction for the formation of MgF_2_ is shown in the following response equation [49]:

(4) $${\mathrm{MgCl}}_{2}{+\mathrm{CaF}}_{2}{=\mathrm{MgF}}_{2}{+\mathrm{CaCl}}_{2}$$

The reaction equilibrium constant of Equation (4), calculated by HSC Chemistry 6.0, is *K*_eq_ = 3.827·10^2^. This reaction also occurs in flux #5. Thus, the reaction corresponds to the endothermic peak shared by fluxes #4 and #5 at 460 °C. The heating curves of fluxes #4 and #5 move in the positive direction of the y-axis after the reaction occurs, indicating that the reaction absorbs a quantity of heat. In the range of 25 °C to 100 °C, KMgCl_3_(H_2_O)_6_ was confirmed to exist in the MgCl_2_–KCl–H_2_O system by Cui et al. [50]. Since it is obtained by heating the mixture of MgCl_2_ and KCl after absorbing water, the reaction might be as follows:

(5) $${\mathrm{MgCl}}_{2}+\mathrm{KCl}+H_{2}O={\mathrm{KMgCl}}_{3}{{(H}_{2}O)}_{6}$$

### 3.2. Physical Properties of Fluxes

#### High-Temperature Contact Angle for Fluxes and Alumina

Based on the data in Table 3 and Figure 1d, fluxes #2, #4, #7, and #10 were selected for high-temperature contact angle testing with alumina in this paper. According to the research of Utigard et al. [24], salts such as chloride and carbonate in the flux will not affect the wettability of the flux, which contributes to the flux grouping of this work. Since only one of the main components in the compositions of fluxes #1, #2, and #3 is different, they were grouped. Fluxes #4 and #5 were grouped because the chloride in the main components is different. Fluxes #6 and #7 were grouped because the main components are different. After all, the main components of flux #6 are chloride and carbonate and #7 is all carbonate. Fluxes #8, #9, and #10, for which the main components are all made up of three kinds of molten salt, were grouped.

**Figure 3:**
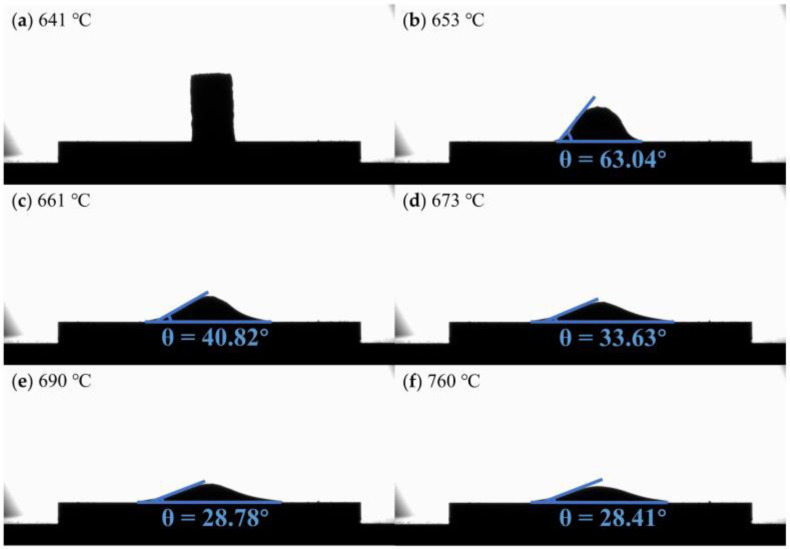
Images of the contact angle between flux #2 and alumina plate at different temperatures: (**a**) 641 °C; (**b**) 653 °C; (**c**) 661 °C; (**d**) 673 °C; (**e**) 690 °C; (**f**) 760 °C.

**Figure 4:**
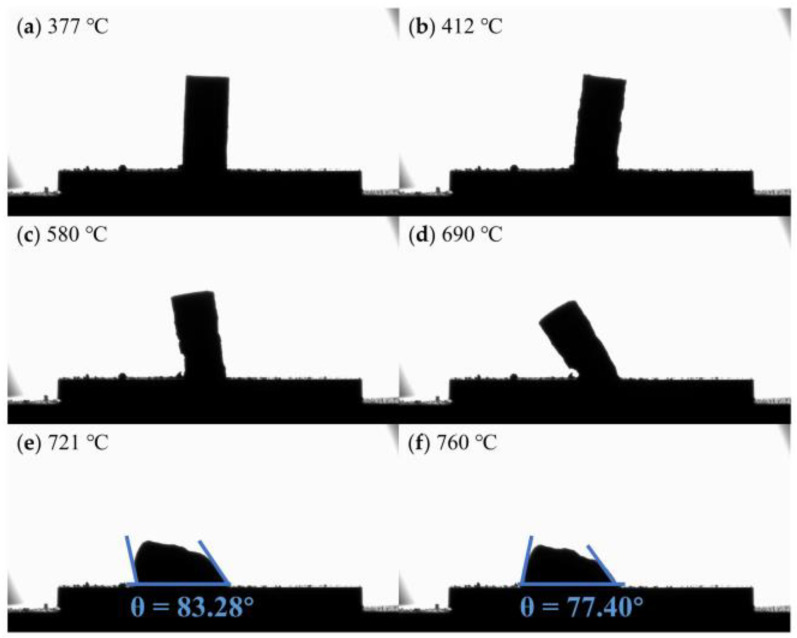
Images of the contact angle between flux #4 and alumina plate at different temperatures: (**a**) 377 °C; (**b**) 412 °C; (**c**) 580 °C; (**d**) 690 °C; (**e**) 721 °C; (**f**) 760 °C.

**Figure 5:**
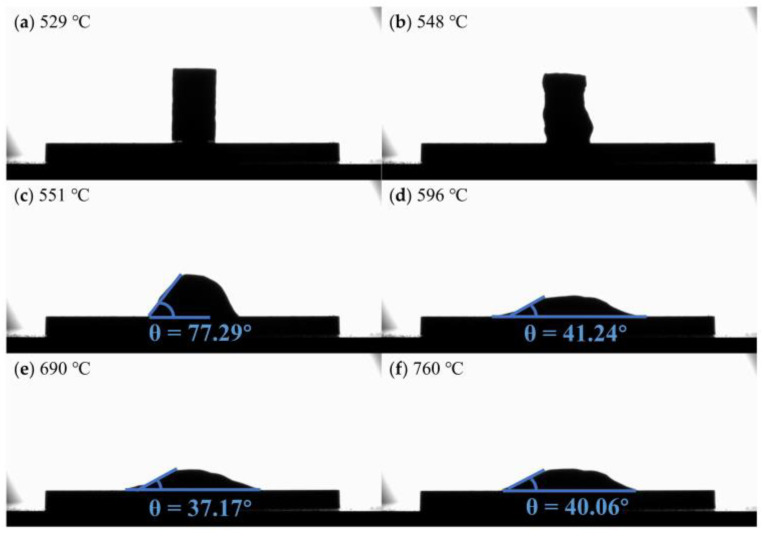
Images of the contact angle between flux #7 and alumina plate at different temperatures: (**a**) 529 °C; (**b**) 548 °C; (**c**) 551 °C; (**d**) 596 °C; (**e**) 690 °C; (**f**) 760 °C.

Figure 3 shows the contact angle of flux #2 and alumina at different temperatures. The temperature at which the sample begins to soften is 653 °C, which is close to the melting point (653.8 °C) measured experimentally, shown in Figure 1d. The contact angle becomes progressively smaller with increasing temperature and reaches a minimum of 28.41° at the refining temperature (760 °C). Figure 4 and Figure 5 show the contact angles of flux #4 and flux #7, with alumina at different temperatures, respectively. The softening temperatures of the samples are both close to the DSC results. Flux #4 has a larger temperature range from the onset of softening to the liquid state, presumably due to the phase change at a high temperature, which increases the solid–liquid phase zone of the mixture and also results in a larger contact angle of the mixture with alumina at the refining temperature compared to the other fluxes.

**Figure 6:**
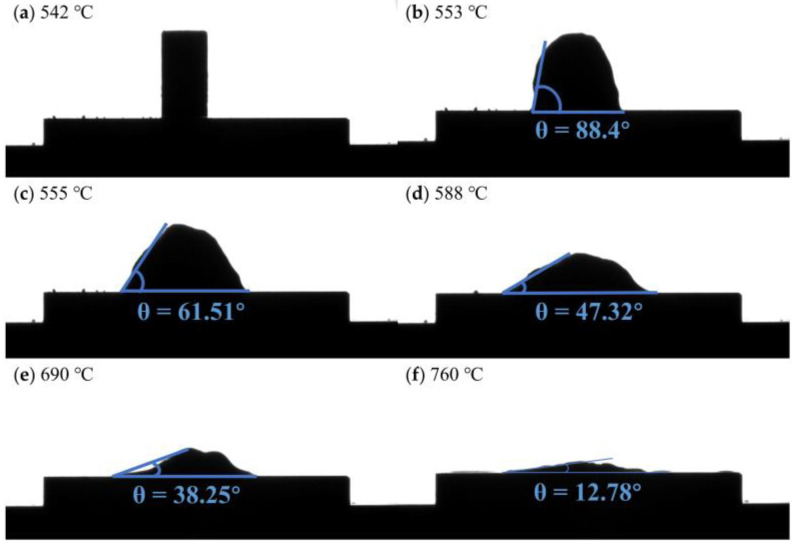
Images of the contact angle between flux #10 and alumina plate at different temperatures: (**a**) 542 °C; (**b**) 553 °C; (**c**) 555 °C; (**d**) 588 °C; (**e**) 690 °C; (**f**) 760 °C.

**Figure 7:**
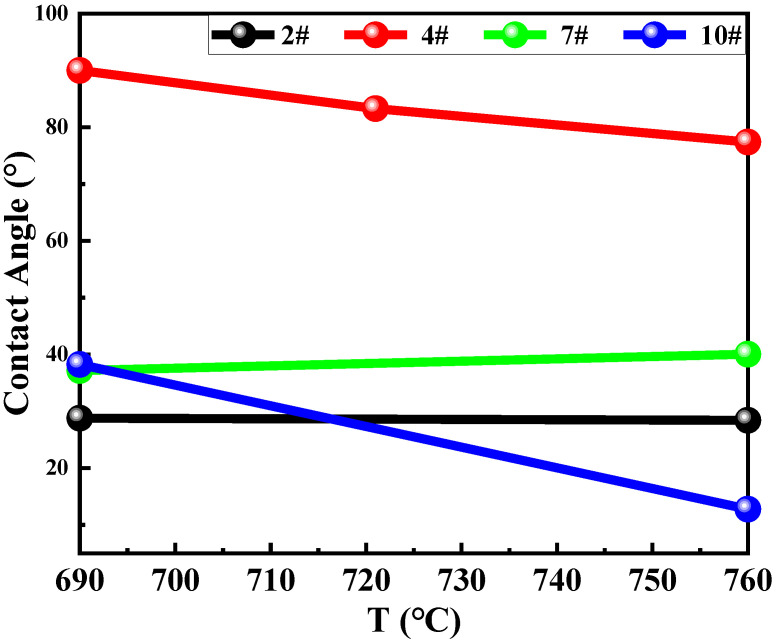
The contact angle of the fluxes with the alumina in the refining temperature range.

Figure 6 shows the contact angle of flux #10 and alumina at different temperatures. The flux did not start melting until 541 °C, and the contact angle with the alumina remained at 90°. At 553 °C the flux started to soften, which was close to the DSC test result (562.2 °C). The error values may be caused by different test conditions and equipment. When flux #10 was heated to 555 °C, the flux started to spread on the alumina ceramic plate, and the contact angle was reduced to 61.51°. Then, as the heating temperature increased, the contact angle between the flux and the alumina showed a trend of gradually decreasing, and the flux also gradually spread out on the surface of the alumina in the image, which also indicated that there was a good wettability between flux #10 and alumina. Then, aluminum alloy is heated to a refining temperature of 760 °C and the contact angle is continuously reduced, where the contact angle changes, as shown in Figure 7, with the contact angle eventually reducing to 12.78°.

Figure 7 shows the variation in contact angle between fluxes and alumina over the aluminum alloy refining temperature range (730–760 °C). At the refining temperature range, flux #4 has the largest contact angle with alumina. Fluxes #2, #7, and #10 all contain Na_2_CO_3_ as the main component, so their contact angle is close, but flux #10 contains NaF. NaF can decrease the contact angle, which correlates with interfacial tensions among the aluminum melt, molten flux, and inclusions [18], accordingly, reducing the contact angle of flux #10 and alumina.

### 3.3. Thermodynamics of Flux Refinement

According to Majidi and Liu et al. [26,51], although the density of oxide inclusions (3.43–3.72 g/cm^3^) are higher than that of molten aluminum (2.3–2.5 g/cm^3^), they float in the melt because of their high area to volume ratio, and can increase their buoyancy by adsorbing hydrogen. Usually, because of the good wettability of the inclusions, and the lower density (1.9–2.1 g/cm^3^) compared to the aluminum melt (2.3–2.5 g/cm^3^) [19,52], fluxes are often used to remove the oxide inclusions by absorbing the inclusions, forming clusters, and then floating up to the surface of the molten aluminum together.

**Figure 8:**
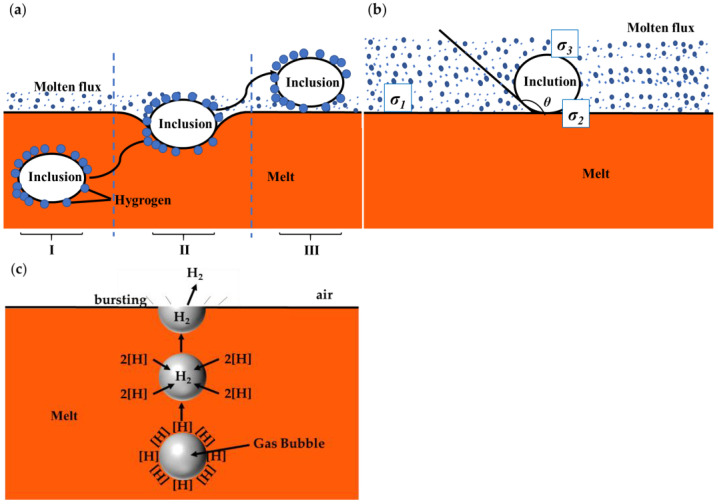
Schematic diagram of inclusions removal and degassing during flux treatment: (**a**) migration of inclusions from melt to flux. stage I: the inclusions approaching the melt–flux interface, stage II: inclusions crossing the aluminum melt–flux interface, stage III: inclusions transferring from the interface to the interior of the molten flux; (**b**) surface tension between inclusions and flux; (**c**) degassing through bubbles produced by the reaction between flux and oxide inclusion.

When the fine inclusions that are floating in the melt are close to the flux interface, the inclusions migrate into the molten flux under the adsorption effect between the two phases, and the adsorption process is roughly divided into three stages, as shown in Figure 8:

Stage 1: the inclusions approaching the melt–flux interface;
Stage 2: inclusions crossing the aluminum melt–flux interface;
Stage 3: inclusions transferring from the interface to the interior of the molten flux.

The process of the inclusions being adsorbed by the flux is shown in Figure 8a. The process of inclusions migrating from alloy melt to flux was analyzed thermodynamically. Assuming that the surface area of the inclusions is *S*, the surface free energy *G*_1_ of the inclusions before they are adsorbed by the flux can be expressed as:

(6) $$G_{1}=S\sigma_{1}+S\sigma_{2}$$

where *σ*_1_ is the surface free energy between the melt and flux, and *σ*_2_ is the surface free energy between melt and inclusions.

When the inclusions migrate into the molten flux, the surface energy of the system is only *G*_2_, which can be expressed as:

(7) $$G_{2}=S\sigma_{3}$$

where *σ*_3_ is the surface free energy between the flux and inclusions.

Then the change in the free energy of the system, Δ*G*, during the migration of the inclusions can be expressed as:

(8) $$\Delta G=G_{2}-G_{1}=S\left(\sigma_{3}-\sigma_{2}-\sigma_{1}\right)$$

The surface tension of the inclusions at the interface between the molten aluminum and the flux is illustrated in Figure 8b.

According to Young’s equation [53]:

(9) $$\cos\left(180{}^{\circ}-\theta \right)=\frac{\sigma_{2}-\sigma_{3}}{\sigma_{1}}$$

After calculation we obtain:

(10) $$\cos\;\theta =\frac{\sigma_{3}-\sigma_{2}}{\sigma_{1}}$$

where *θ* is defined as the equilibrium contact angle between the molten aluminum and the inclusions.

Because there is no wetting between the inclusions and the melt, the contact angle is *θ* > 90°, so cos *θ* < 0, and since all surface tensions are known to be positive, we obtain:

(11) $$\sigma_{3}-\sigma_{2}\;<\;0$$

Combining Equations (8) and (11), it can be concluded that Δ*G* < 0. The experiments show that the flux and the oxide inclusions have good wettability, from which it can be seen that the oxide inclusions can be spontaneously adsorbed by the flux at the refining temperature.

According to the studies of Li [18] and Bonn et al. [54], the driving force of flux spreading on the inclusions, before the flux wetting reaches equilibrium, comes from the unbalanced interfacial tension, which is determined as:

(12) $$I_{t}=\sigma_{1}\left(\cos\left(180\;-\;\theta \right)-\;\cos\theta_{t}\right)$$

where *θ_t_* is the time-dependent dynamic contact angle, which is determined as [28,55]:

(13) $$V=\frac{\pi R^{3}{}_{\left(t\right)}\left(2\;-\;3\cos\theta_{t}+\cos^{3}\theta_{t}\right)}{3\sin^{3}\theta_{t}}$$

where *V* is the volume of the molten flux droplet (defined as constant), and *R*_(*t*)_ is the time–dependent base radius of the droplet, which is in the shape of a spherical cap [18]. According to Equations (9), (12) and (13), through increasing the size of flux powders (i.e., the volume of molten flux droplets at the refining temperature), and adding NaF into the flux (i.e., decreasing *θ*, which correlates with interfacial tensions among the three phases), it is possible to achieve a greater driving force for spreading, and realize a wetting mechanism transition from adsorbing to engulfing, which will then respectively improve the efficiency of wetting and rate of floatation of a molten flux droplet. In consequence, as shown in Figure 6 and Figure 7, flux #10 has a better spreading effect and a smaller contact angle, because the addition of NaF in flux #10 results in a reduction in surface tension between flux #10 and the inclusions. In addition, Fu et al. [9] indicated that the removal of oxide inclusions is important to eliminate pores, because part of the hydrogen will accumulate and adsorb around the inclusions, as shown in Figure 8a. The hydrogen that accumulates around the inclusions, and is adsorbed by the inclusions, could be removed along with the removal of inclusions. The hydrogen could be adsorbed in the inclusions, which could also be eliminated through the inclusions’ removal. However, there is still some dispersive hydrogen within the aluminum melt, which is removed by the alternative scheme shown in Figure 8c. When the flux is added to the aluminum melt, some components of the flux react, to form bubbles, and the hydrogen that is dissolved in the aluminum melt diffuses into the bubbles. Then, it is removed from the aluminum melt with the bubbles. That is the reason we present the degassing scheme in Figure 8c.

It is assumed that the concentration of hydrogen in the aluminum melt at any given moment is [PctH], the equilibrium concentration of hydrogen on the bubble surface is [PctH]_e_, the activity coefficient of hydrogen is *f*_H_, the partial pressure of hydrogen inside the bubble is *P*_H2_, and the equilibrium constant is *K*, that is temperature dependent only. The hydrogen in the aluminum melt reacts at the surface of the bubbles as follows [56]:

(14) $$[H]=\frac{1}{2}H_{2}$$

and the following condition is satisfied when Equation (14) reaches chemical equilibrium:

(15) $$\frac{{\left[\mathrm{PctH}\right]}_{e}f_{H}}{K}=\sqrt{P_{H_{2}}}$$

The driving force for the diffusion of hydrogen at any given moment is the difference between the hydrogen concentration in the melt and the hydrogen concentration at the interface between the liquid and gas phases. The [H] in the melt diffuses through the boundary layer between the liquid and gas phases to the surface of the bubble, leading to an increase in hydrogen concentration at the interface, and the hydrogen is generated on the bubble surface and diffuses into the bubble rapidly. With the diffusion of hydrogen in the boundary layer, the hydrogen is formed at the bubble surface, and diffuses to the inside of the bubble rapidly, increasing the partial pressure of hydrogen inside the bubble. Until the interface reaches chemical equilibrium, and satisfies [PctH] = [PctH]_e_, the driving force for diffusion and the difference in partial pressure of hydrogen between the inside and outside of the bubble is zero, and the hydrogen that is formed at the interface can no longer diffuse into the bubble. Finally, the bubbles float up to the surface of the aluminum melt and burst. According to the studies of Utigard et al. [19], Na_2_CO_3_ in the flux will form bubbles within the aluminum melt by the following reaction, and remove the hydrogen that is dissolved in the aluminum melt [57]:

(16) $${\mathrm{Na}}_{2}{\mathrm{CO}}_{3\;}+{\mathrm{Al}}_{2}O_{3}=2{\mathrm{NaAlO}}_{2}+{\mathrm{CO}}_{2}$$

### 3.4. The Results of Flux Refinement

**Figure 9:**
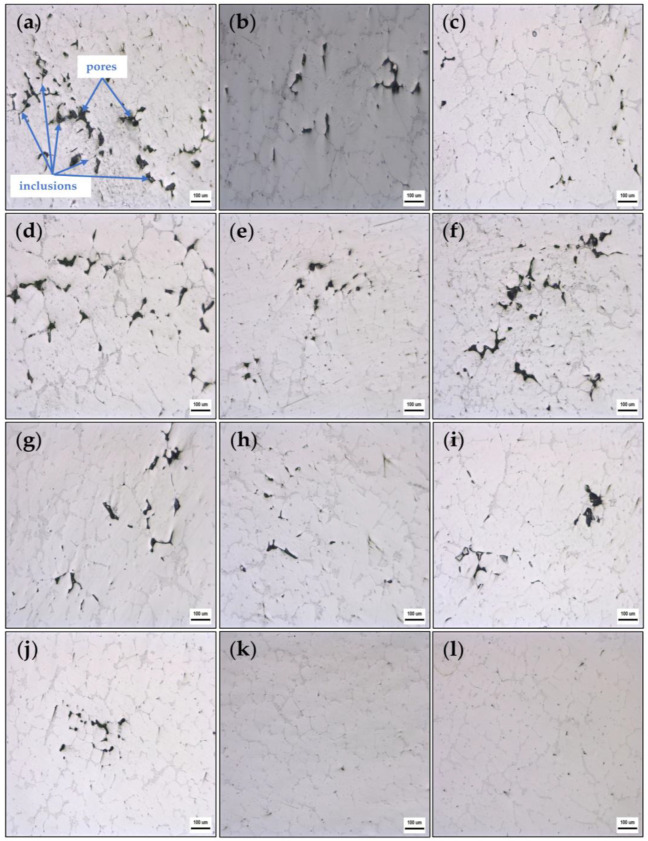
Microstructure of the samples: (**a**) unrefined; (**b**) refined by #1; (**c**) refined by #2; (**d**) refined by #3; (**e**) refined by #4; (**f**) refined by #5; (**g**) refined by #6; (**h**) refined by #7; (**i**) refined by #8; (**j**) refined by #9; (**k**) refined by #10; (**l**) refined by flux STJ–A3.

Figure 9 shows the microstructures of the AA6111 alloy before and after flux refinement, and the pores and inclusions content before and after flux refinement.

Figure 9a shows a metallographic photograph of a cast sample of untreated AA6111 aluminum alloy. Numerous pores and inclusions can be seen. The pores are nearly 100 µm in diameter and irregularly distributed. According to Wang’s [58] research, aluminum oxide inclusions within the aluminum melt often show an amorphous state, in the form of strings and films. After the melt cooling, the oxide formed stripe-like inclusions, as shown in Figure 9a. In addition, the aluminum oxide cluster makes the ambient Al atoms disordered in the aluminum melt, and creates more vacancies to accumulate hydrogen, which causes the formation of pores in the aluminum [9]. Thus, inclusions in the form of strings and films are associated with concomitant pores, as shown in Figure 9a–j.

**Figure 10:**
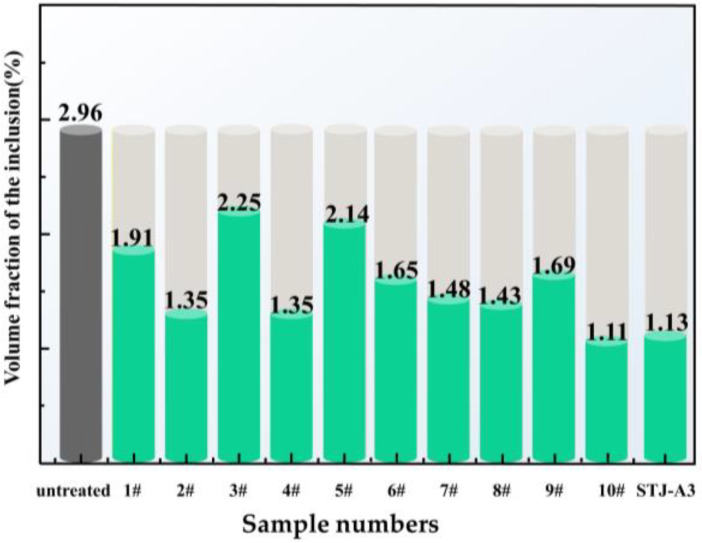
The pores and inclusions contents of untreated, fluxes #1–#10 and STJ-A3 refined samples.

Figure 9b–d show the microstructure of AA6111 aluminum alloy refined by fluxes #1, #2, and #3, respectively. It can be seen that the pores in Figure 9b are significantly smaller and more uniform in size than in Figure 9a, and there are fewer inclusions than before the refinement. Only a few small inclusions remain, which are also more evenly distributed than before the flux refinement. Compared to the large area and size of the pores and inclusions in Figure 9a, the pores and inclusions in Figure 9c, after refining with flux #2 have been significantly removed, with the pores and inclusions being smaller and less concentrated, and more evenly distributed throughout the alloy. After melting, NaF formed in flux #2 reduced the interfacial tension between the flux and inclusions, and reduced the contact angle between flux #2 and the inclusions. In addition, flux #2 contains 51.8wt.%Na_2_CO_3_, which will react as in Equation (16), to form CO_2_ bubbles and remove the hydrogen. Thus, compared with fluxes #1 and #3, the result of refinement (as shown in Figure 10) was better. The pores and inclusions of the sample after refining were reduced to 1.35%. A comparison shows that the number and size of pores in Figure 9d are slightly improved compared with Figure 9a, but the improvement is limited and the reduction in inclusions is more pronounced than the pores.

Figure 9e,f shows the metallograph of a cast sample after refining the melt with flux #4 and flux #5, respectively. It can be seen that the pores and inclusions in Figure 9e are significantly reduced compared to the sample without any flux refining (shown in Figure 9a), which shows the pores and inclusions are significantly smaller in size, averaging around 20 µm and 5 µm, respectively. The sample treated by flux #5 (shown in Figure 9f) is similar to the result using flux #3 (shown in Figure 9d), with the pores appearing more concentrated in the flux #5-treated sample, but slightly less numerous than in Figure 9d. Although the contact angle between flux #4 and aluminum oxide is large, about 80° (shown in Figure 7), fluxes #4 and #5 contain MgF_2_. This can react in the molten aluminum as follows [24]:

(17) $${\mathrm{MgF}}_{2}+\mathrm{Al}={\mathrm{AlF}}_{3}+\mathrm{Mg}$$

(18) $${\mathrm{AlF}}_{3}+\mathrm{Na}/\mathrm{Ca}=\mathrm{NaF}/{\mathrm{CaF}}_{2}+\mathrm{Al}$$

This compensates for the poor wettability of flux #4 with oxide at the refining temperature, and improves the refining effect of flux #4. In addition, flux #4 contains MgCl_2_ and KCl, a combination that has good cover properties [19] and prevents the aluminum melt from absorbing hydrogen. After refining, the pores and inclusions content of the sample is reduced to 1.35% (shown in Figure 10).

Figure 9g,h shows the metallograph of a cast sample after refining by flux #6 and flux #7, respectively. In Figure 9g, the inclusions were significantly reduced after being refined by flux #6, and the size and number of pores were also significantly smaller and less concentrated than those in the sample without any flux refinement. It can be seen that the pores and inclusions are significantly reduced and the size is reduced to around 10–20 µm, compared to Figure 9a. Within the refining temperature range (730–760 °C), the contact angle between flux #7 and aluminum oxide is about 40°, which indicates that the flux can absorb inclusions well in the melt. The composition of flux #7 contains 46.5 wt.% Na_2_CO_3_ and 40.5 wt.% K_2_CO_3_, and the composition of flux #6 contains 46.3 wt.% K_2_CO_3_. According to Utigard et al. [19,24], K_2_CO_3_ and Na_2_CO_3_ can form CO_2_ bubbles in the aluminum melt and thus remove hydrogen. The pores and inclusions content of the sample after refining is reduced to 1.48% (as shown in Figure 10).

Figure 9i–k show the microstructure of the AA6111 aluminum alloy refined by fluxes #8, #9, and #10, respectively. The effect of the refinement using flux #8 is similar to that using flux #7, although flux #8 is better at purifying inclusions. It can be seen that the pores and inclusions in Figure 9j are more significantly reduced and the size is smaller than those before the refinement by flux #9. As can be seen in Figure 9k, the number of pores and inclusions in the sample is reduced and the size is not as large as in Figure 9a, and the size of the pores and inclusions is only about 5 µm, or smaller, and evenly distributed. In the group of fluxes including #8, #9, and #10, the best refining performance is from flux #10 (as shown in Figure 10). At the refining temperature (760 °C), the contact angle between flux #10 and aluminum oxide is 12.78°. Its good wettability with inclusions is due to the composition containing 11.0 wt.%NaF. In addition, 29.5 wt.%NaCl is present in flux #10, which makes it easy to separate the flux from the aluminum melt. The Na_2_CO_3_ (46.5 wt.%Na_2_CO_3_) in the flux can react (Equation (16)) to form CO_2_ bubbles and remove the hydrogen, as shown in the scheme in Figure 8c. AA6111 alloy treated with flux #10 has the highest effect of refinement. After refining, the pores and inclusions content of the sample is reduced to 1.11% (as shown in Figure 10), and the inclusions and pores removal rate reaches 62.5%.

Figure 9l shows the microstructure of the alloy refined by the STJ–A3 flux. It can be seen that the pores have reduced significantly in the sample, and only retained some fine and slender inclusions. According to the results in Figure 10, the refining effect of the STJ–A3 flux is better than all the rest of the fluxes, and only slightly worse than flux #10.

From the results of this work, it can be concluded that the refining efficiency of different fluxes is highly dependent on their chemical composition, melting temperature, and wettability with aluminum oxide, which agrees with the conclusions of Gyarmati et al. [15]. The ten fluxes in this work all have significantly lower melting temperatures than the refining temperature, which is a prerequisite to enable the fluxes to have a refining effect in the melt. For the molten salt-based fluxes (fluxes #1–#7), fluxes #2 and #7 show better refining effects, because they contain two exothermic components. This is because the refining effect is determined by the coupling of the degassing and inclusions removing effects. The Na_2_CO_3_ and K_2_CO_3_ in fluxes #2 and #7 can react in the melt to form bubbles, removing some dispersive hydrogen dissolved in the aluminum melt. Thus, if the flux is used to refine alloys with a high hydrogen content, the content of components among which the exothermic reaction occurs should be increased accordingly. After comparing the refining effects of fluxes #1, #3, #4, and #5, it was found that fluxes #3 and #5, which contained NaCl, were not as effective as fluxes #1 and #4, which contained KCl. For the three molten salt-based fluxes, #8, #9, and #10, the refining effect of flux #10, which contained NaF, was better than that of flux #8, which contained KCl. Thus, if the flux is used to refine alloys with a high inclusions content, attention should be paid to the contact angle between the flux and the oxide [19,24], and molten salt, that improves the wettability of the flux and inclusions, could be added as appropriate.

## 4. Conclusions

A three-step strategy for flux composition design was presented in this paper. The composition ratio of various fluxes was designed by using eutectic point components of different molten salt systems, and the main components of ten different fluxes were designed. The melting points of these fluxes were investigated using DSC, and the phase changes of the fluxes were investigated experimentally using XRD. The high-temperature wettability of the fluxes and alumina was characterized, and the process of the fluxes removing inclusions and degassing was analyzed thermodynamically. Finally, the fluxes refinement effectiveness on the microstructure of the AA6111 alloy was investigated. In addition, the refining effect of the fluxes was compared with that of the flux STJ–A3, currently used in practice. The experimental results show that the flux composition design method proposed in this study is feasible, and a flux with the best refining effect can be preferentially selected, which will not be limited to the design of a few components. This work will not only provide the thermodynamic basis and experimental data for establishing a database of aluminum alloy flux design in the future, but also provide a strategy for screening more components for flux design, which can reduce the workload of flux design. The conclusions can be drawn as follows:

(1) The melting points of all ten fluxes were lower than the refining temperature (760 °C). The flux with the highest melting point was flux #9, with a melting point of 660 °C. The lowest melting point was flux #4, at 412.1 °C.
(2) Among the 10 fluxes, the phases of fluxes #2, #4, and #5 changed significantly after melting, with the main components of flux #2 changing from Na_2_CO_3_ and Na_2_SO_4_ to NaF, Na_2_CO_3_, and Na_3_FSO_4_. Flux #4 changed from KCl and MgCl_2_ to MgF_2_ and KMgCl_3_(H_2_O)_6_.
(3) The contact angles between all four groups of fluxes and aluminum oxide at the refining temperature (760 °C) were less than 90°, proving that they were all capable of wetting with oxide inclusions at the refining temperature; flux #10 had the smallest contact angle of 12.78°. The thermodynamic analysis confirms that the flux can spontaneously adsorb inclusions within the aluminum melt.
(4) All the fluxes can refine AA6111 alloy melt, and flux #10 has the best effect, with a removal rate of 62.5%. After refining by flux #10, the alloy microstructure is significantly cleaner than before refinement, with the pores and inclusions content of the sample reduced to 1.11% from 2.96%. The refinement effectiveness is even better than the flux STJ–A3.
(5) The refining efficiency of different fluxes is highly dependent on their chemical composition, melting temperature, and wettability with aluminum oxide. The flux has a significantly lower melting temperature than the temperature of refinement is a prerequisite to enable the fluxes to have a great refining effect in the melt. When the flux is used for refining alloys with a high hydrogen content, the exothermic component of the flux should be increased accordingly. When the flux is used for refining alloys with high oxide inclusions, components that improve the wettability of the flux and inclusions should be added appropriately.

## Figures and Tables

**Table 1:** Thermophysical parameters of salt compounds used in fluxes.

Chemicals	Molecular Mass (g/mol)	Solid Density (g/cm^3^)	Melting Point (°C)	Boiling Point (°C)
LiCl	43.39	2.068	605	1325
NaCl	58.44	2.165	801	1413
KCl	74.56	1.984	770	1500
CaCl_2_	110.99	2.15	782	1600
MgCl_2_	95.22	2.32	714	1412
AlCl_3_	133.34	2.44	190	177.8
BaCl_2_	208.25	3.92	963	1560
LiF	25.94	2.635	845	1676
NaF	41.99	2.558	993	1695
KF	58.1	2.48	858	1505
CaF_2_	78.08	3.18	1423	2500
MgF_2_	62.31	3.18	1261	2239
AlF_3_	83.98	2.882	–	1291
Na_3_AlF_6_	209.94	2.9	1010	–
LiNO_3_	68.94	2.38	264	600
NaNO_3_	84.99	2.261	307	380
KNO_3_	101.11	2.109	339	400
Li_2_SO_4_	109.94	2.221	859	high
Na_2_SO_4_	142.04	–	897	–
K_2_SO_4_	174.27	2.66	1069	1689
CaSO_4_	136.14	2.61	1450	high
MgSO_4_	120.37	2.66	–	1124
Na_2_CO_3_	105.99	2.532	851	high
K_2_CO_3_	138.21	2.42	894	high
MgCO_3_	84.32	2.96	–	350
CaCO_3_	100.09	2.71	1339	850

**Table 2:** The eutectic point compositions and eutectic temperatures of selected commonly used fluxes.

System	Eutectic Points Concentration (mol%)	Eutectic Temperature (°C)	Ref.
KCl: Na_2_CO_3_	49.6: 50.4	581.76	[35]
Na_2_SO_4_: Na_2_CO_3_	37.0: 73.0	626	[36]
NaCl: Na_2_CO_3_	54.6: 45.4	632	[37]
KCl: MgCl_2_	63.5: 36.5	445	[38]
NaCl: MgCl_2_	58.5: 41.5	428	[38]
KCl: K_2_CO_3_	62.0: 38.0	623	[39]
Na_2_CO_3_: K_2_CO_3_	60.0: 40.0	713	[39]
Na_2_CO_3_: NaCl: KCl	31.0: 34.0: 35.0	573	[39]
NaF: NaCl: NaNO_3_	5.0: 8.0: 87.0	288	[40]
NaF: NaCl: Na_2_CO_3_	21.6: 41.9: 36.5	576	[41]
CaF_2_: Na_3_AlF_6_	50: 50	945.5	[42]

**Table 3:** Chemical compositions of experimental fluxes.

Sample Numbers	Compositions (wt.%)									
	KCl	Na_2_SO_4_	Na_2_CO_3_	NaCl	MgCl_2_	K_2_CO_3_	NaF	NaNO_3_	CaF_2_	Na_3_AlF_6_
#1	43.1	–	43.8	–	–	–	–	–	3.0	10.0
#2	–	35.2	51.8	–	–	–	–	–	3.0	10.0
#3	–	–	52.3	34.7	–	–	–	–	3.0	10.0
#4	50.7	–	–	–	36.3	–	–	–	3.0	10.0
#5	–	–	–	40.4	46.6	–	–	–	3.0	10.0
#6	40.7	–	–	–	–	46.3	–	–	3.0	10.0
#7	–	–	46.5	–	–	40.5	–	–	3.0	10.0
#8	28.8	–	36.4	21.8	–	–	–	–	3.0	10.0
#9	–	–	–	5.0	–	–	2.3	79.7	3.0	10.0
#10	–	–	46.5	29.5	–	–	11.0	–	3.0	10.0

**Table 4:** Chemical composition of AA6111 aluminum alloy.

Elements	Al	Si	Mn	Mg	Fe	Zn	Ti	Cr	Cu
Mass fraction (wt.%)	Bal.	0.85	0.25	0.73	0.38	0.13	0.08	0.07	0.58

## Data Availability

Data supporting reported results can be found in this study.
